# A Single-Center Experience on HLA Typing with 11 Loci Next Generation Sequencing in Korean Patients with Hematologic Disease

**DOI:** 10.3390/diagnostics12051074

**Published:** 2022-04-25

**Authors:** Namsoo Kim, Sinyoung Kim, Jong Rak Choi, Younhee Park

**Affiliations:** Department of Laboratory Medicine, Severance Hospital, Yonsei University College of Medicine, Seoul 03722, Korea; shaun0912@yuhs.ac (N.K.); sykim@yuhs.ac (S.K.); cjr0606@yuhs.ac (J.R.C.)

**Keywords:** human leukocyte antigen, HLA typing, next generation sequencing, hematologic malignancy

## Abstract

The human leukocyte antigen (*HLA*) system comprises the most polymorphic genes of the human genome and is famous for its potential pathological roles. To accurately type HLA genes and find HLA-matched donors, which are critical for effective hematopoietic transplantation, HLA typing using next-generation sequencing (NGS) was implemented. We aimed to share the experience of HLA typing using NGS in patients with hematologic malignancies and evaluate its association with hematologic diseases. Data from 211 Korean, non-familial patients diagnosed with a hematologic disease were reviewed, and NGS was performed for 11 *HLA* loci. Three-field HLA typing with G code was successfully achieved for all loci and the known linkage between *HLA-DRB3/4/5* and *HLA-DRB1* was fully matched. Therefore, NGS-based HLA typing enables a detailed, high-resolution analysis of the HLA system that can help with the selection of suitable donors. Notably, HLA-DRB1*08:02:01G was significantly associated with myelodysplastic syndrome. Although this result confirms the tendency of some alleles to be associated with hematological disorders, this may not be the case in hematologic malignancies. Nonetheless, NGS-based HLA typing data for *HLA-DP*, *HLA-DQ*, and *HLA-DRB3*/4/5 are still warranted for a better understanding of the corresponding locus.

## 1. Introduction

The human leukocyte antigen (HLA) complex comprises the most polymorphic genes of the human genome, which are widely recognized for being associated with various disease states [1]. Accurate and precise HLA typing is important in solid organ and hematopoietic transplantations for HLA-matched donor selection. Molecular HLA typing methods, such as sequence-specific oligonucleotide probes and primers, or Sanger-based sequence-based typing, have been commonly used in clinical laboratories. However, these methods are known to provide ambiguous results as they analyze few exons, but also, in some cases, can deliver erroneous results owing to their inability to detect variants of targeted exons [2]. To overcome these limitations, next generation sequencing (NGS) was introduced and established to ensure accurate HLA typing by sequencing the entire exons and introns of HLA genes [3].

HLA matching in hematopoietic stem cell transplantation (HSCT) requires high resolution or more than 2-field HLA typing of *HLA-A*, *HLA-B*, *HLA-C*, *HLA-DRB1*, and *HLA-DPB1* [4], for which the above-mentioned classical methods may not be suitable. Several studies have explored NGS for the analysis of these genes [5,6], but NGS data on *HLA-DPA1*, *HLA-DPB1*, *HLA-DQA1*, and *HLA-DRB3/4/5* remain scarce. Treating malignant hematologic disease, which has a prevalence of >63 cases per 100,000 individuals, has remained challenging, and allogenic HSCT using an HLA-matched unrelated donor is now one of the best choices, especially in patients with intermediate or unfavorable risk of acute myeloid leukemia [7]. Thus, the demand for HSCT and to find an appropriate HLA-matched donor via HLA typing is increasing. Prior to typing HLA using NGS, Sanger-based typing (SBT) was the standard approach. Because SBT is limited by its ambiguity and NGS has the strength of a very low error rate, the use of NGS-based HLA typing is increasing in HSCT. It has been shown that the accurate matching of *HLA-A*, *HLA-B*, *HLA-C*, *HLA-DR*, *HLA-DP*, and *HLA-DQ* in ultra-high resolution may increase the probability of overall survival [8]. In addition, it has been suggested that matching those 12 alleles with ultra-high resolution may reduce acute graft-versus-host disease risk [9]. These studies showed that matching 12 alleles is important for patients who undergo HSCT, also increasing demand for accurate HLA typing results and more *HLA* locus.

Several reports have described the association between HLA and hematologic diseases. For example, *HLA-C3* is associated with acute myeloid leukemia in the Korean population [10], HLA-B*40 is associated with acute lymphoblastic leukemia in the Mexican population [11], and HLA-B*40:02 is associated with acquired aplastic anemia in Japanese patients [12]. Here, we describe our HLA typing results using NGS in patients diagnosed with hematologic disease. We also investigated the association of HLA with hematologic malignancies and compared HLA frequencies with those of the general Korean population.

## 2. Materials and Methods

### 2.1. Sample Collection

Clinical data were collected from the clinical records of 211 patients who underwent high-resolution HLA typing for HSCT between July 2019 and May 2021. The study was conducted according to the guidelines of the Declaration of Helsinki, and its protocol was reviewed and approved by the Institutional Review Board (IRB) of the Severance Hospital in Seoul, Korea (IRB Number: 4-2021-1249). The need for informed consent was waived due to the retrospective nature of the study (review of medical records) on the condition that the study involved no more than minimal risk to the patients and that their privacy was thoroughly protected.

### 2.2. HLA Typing

The AllType NGS 11-Loci Amplification Kit (One Lambda, West Hills, CA, USA) was used to amplify target DNA regions. Whole blood samples (3 mL) were collected in an ethylenediaminetetraacetic acid-treated tube, DNA was extracted using QIASymphony (Qiagen, Hilden, Germany), and its final concentration was adjusted to 25 ng/μL. A mixture of reagents was prepared according to the number of samples and pipetted into a polymerase chain reaction (PCR) plate containing DNA template. The PCR primers were added and cycling conditions were set according to the manufacturer’s instructions as follows: 1 cycle of denaturation at 94 °C for 2 min, 30 cycles of 10 s at 98 °C, 3 min at 69 °C. DNA amplicons were purified by mixing Agencourt AMPure XP Reagent (Beckman Coulter, Brea, CA, USA), quantified using a Qubit 3.0 fluorometer and Qubit dsDNA HS Assay kit (Thermo Fisher Scientific, Waltham, MA, USA) according to the manufacturer’s instructions, and diluted to ensure equal concentrations. The amplicons were fragmented using the Ion Shear Plus Reagents Kit (Thermo Fisher Scientific) and then ligated using the Ion Plus Fragment Library Kit (Thermo Fisher Scientific) in each well. Next, size selection using the Agencourt AMPure XP Reagent (Beckman Coulter) was performed. Secondary amplification was performed using Platinum PCR Supermix high Fidelity (Thermo Fisher Scientific) and AllType NGS Library Primer mix (One Lambda), with the following PCR cycling conditions: 1 cycle at 95 °C for 5 min followed by 8 cycles of 15 s at 95 °C, 15 s at 58 °C, and 1 min at 70 °C. Amplicon purification and quantification were repeated. AllType NGS Library Pooling Calculator Excel file (One Lambda) was used for library pooling. The obtained library was then sequenced using the Nextseq 550Dx System (Illumina, San Diego, CA, USA). *HLA-A*, *HLA-B*, *HLA-C*, *HLA-DQA1*, and *HLA-DPA1* were fully covered, whereas *HLA-DRB1*, *HLA-DRB3/4/5*, *HLA-DQB1*, and *HLA-DPB1* were only sequenced from exon 2 to the 3′-untranslated region. Sequences were analyzed using the TypeStream Visual NGS analysis software (version 2.1.0.40; One Lambda) and the IPD-IMGT/HLA Database (version 3.40.0.1; https://www.ebi.ac.uk/ipd/imgt/hla/ (accessed on 1 September 2021)). Analysis parameter settings are shown in Table S1.

### 2.3. Statistical Analysis

Microsoft Office Excel (Microsoft Co., Redmond, WA, USA) was used for all statistical analyses. HLA frequencies were calculated and compared with known published data [5,6] or the HLA database created by the Severance Hospital. Statistical significance was set at *p* < 0.05. Differences between any two groups were calculated using the Student’s *t*-test.

## 3. Results

### 3.1. Demographic Characteristics

The demographic features of the patients diagnosed with hematologic diseases are shown in Table 1. Among the 211 patients included in the study, 123 (58.3%) were male and 88 (41.7%) were female. Most patients were aged between 41 and 60 years (38.9%), and the remaining patients were similarly distributed within the other age groups. Major hematological diagnoses were reviewed for all patients, which included acute myeloid leukemia (AML), B-cell acute lymphoblastic leukemia (BLL), myelodysplastic syndrome (MDS), aplastic anemia (AA), T-cell lymphoma (TCL), and chronic myeloid leukemia (CML).

### 3.2. Success Rate of HLA Typing

Table 2 shows the 2-, 3-, and 4-field success rates of typing for each *HLA* locus. Two field HLA typing was completely successful for *HLA-A*, *HLA-B*, *HLA-C*, *HLA-DPA1*, *HLA-DQB1*, *HLA-DRB1*, and *HLA-DRB3/4/5*. However, typing of *HLA-DPB1* and *HLA-DQA1* failed in some samples, achieving 99.10% and 96.83% success rates, respectively. Similar success rate results were obtained for 3-field HLA typing. In contrast, 4-field HLA typing results varied according to each *HLA* locus, successfully accomplishing more than 70% success rate for *HLA-A*, *HLA-B*, *HLA-C*, and *HLA-DQA1*; in particular, *HLA-A* showed the highest success rate at 99.10%. Nevertheless, the success rates of the 4-field typing of *HLA-DRB1* and *HLA-DRB3/4/5* were of 64.93% and 54.89%, respectively, and those of *HLA-DPA1*, *HLA-DPB1*, and *HLA-DQB1* were lower than 30%. Notably, rare alleles that could not be 4-field typed were still regarded as successful by both the 2- and 3-field approaches.

### 3.3. HLA Typing Results

The HLA frequencies identified in the patients diagnosed with hematologic diseases were compared with those of the general population. HLA frequencies were also evaluated and compared based on the different hematologic diseases (Tables S2–S10). G codes were used for reporting ambiguous allele typing. *HLA* alleles that had identical nucleotide sequences across the exons encoding the peptide binding domains were designated by an upper case ‘G’ that follows 3-fields of the allele designation of the lowest numbered allele in the group.

Table 3 shows a summary of the typing results of *HLA-A*, *HLA-B*, and *HLA-C*. Since the published data [6] and/or the data from the HLA database created by the Severance Hospital was only available in two-field resolution or did not completely succeed in four-field typing, we summarized our typing results with two-field resolution. Overall, *HLA-B* was the most diverse gene in our study and in the publicly available data.

Table 4 shows a summary of the two-field typing results of *HLA-DRB1*, and *DQB1*. Other HLA alleles in MHC class II could not be compared because of the lack of reference data. We used the data of In et al. [5] to compare the prevalence of *HLA-DQB1*. Overall, *HLA-DRB1* was more diverse than *HLA-DQB1* in our study and the publicly available data.

*HLA-A* typing results are summarized in Table S2. HLA-A*24:02:01:01 was the most frequent allele in both previously reported Korean population data [6] and this study. HLA-A*02:01:01:01, HLA-A*33:03:01:01, and HLA-A*24:02:01:01 were observed with high frequency. We suggest that the previously reported HLA-A*33:03 group with high frequency in the Korean population matches the HLA-A*33:03:01:01 allele herein reported.

*HLA-B* typing results are summarized in Table S3. *HLA-B* alleles showed the most variability. The three most frequent alleles were HLA-B*15:01, HLA-B*44:03, and HLA-B*51:01 according to Severance data (10.17%, 9.57%, and 9.77%, respectively). Most of those that typed as HLA-B*15:01 referred to HLA-B*15:01:01:01; however, those that typed as HLA-B*44:03 were divided into HLA-B*44:03:01:10, HLA-B*44:03:01G, HLA-B*44:03:02:01, and HLA-B*44:03:02G, and those that typed as HLA-B*51:01 were divided into HLA-B*51:01:01:01, HLA-B*51:01:01:36, and HLA-B*51:01:01G. The most dominant *HLA-B* allele in the Korean population was HLA-B*15:01:01:01.

*HLA-C* typing results are summarized in Table S4. HLA-C*01:02 was the most frequent allele according to previously reported data [6]. In this study, HLA-C*01:02 was divided into HLA-C*01:02:01:01, HLA-C*01:02:01:05, HLA-C*01:02:01:08, and HLA-C*01:02:01G. HLA-C*01:02:01:01 was the most frequent allele in this study, suggesting that the previously reported HLA-C*01:02 group with the highest frequency in Koreans matches the HLA-C*01:02:01:01 allele.

*HLA-DRB1* typing results are summarized in Table S5. HLA-DRB1*04:05, HLA-DRB1*09:01, and HLA-DRB1*15:01 were the three most frequent alleles in the Severance database. In the present study, HLA-DRB1*04:05 was divided into HLA-DRB1*04:05:01:01 and HLA-DRB1*04:05:01:04. HLA-DRB1*09:01 was typed as HLA-DRB1*09:01:02G. The HLA-DRB1*15:01 group was divided into five four-field and one three-field G groups that represented an exact HLA-DRB1*15:01:01:03 match to the previously reported Korean four-field typing.

*HLA-DPA1* is known to have the lowest allele variability among all HLA genes, which was also observed in this study. The success rate of 4-field typing was relatively low, as mentioned above. HLA-DPA1*01:03:01G and HLA-DPA1*02:02:02G had remarkably high frequencies (38.24% and 45.25%, respectively). Detailed information is available in Table S6.

*HLA-DPB1* had the least successful 4-field HLA typing; nonetheless, two 3-field G groups were found to have dominant high frequency—HLA-DPB1*02:01:02G and DPB1*05:01:01G (26.26% and 36.07%, respectively). Other major *HLA-DPB1* alleles in the Korean population were HLA-DPB1*04:01:01G, HLA-DPB1*04:02:01G, and HLA-DPB1*13:01:01G. Detailed information is available in Table S7.

Four-field typing of *HLA-DQA1* showed that HLA-DQA1*03:03:01:03 was the most frequent (11.21%) allele. Other various types of *HLA-DQA1* alleles were detected, including rare allele types, such as HLA-DQA1*05:07, HLA-DQA1*05:08, and HLA-DQA1*05:09. Detailed information is available in Table S8.

Regarding *HLA-DQB1*, the four- and three-field G group results were classified into 15 alleles previously reported in the Korean population. There was correspondence in statistical tendencies. Detailed information, including comparison with previously reported Korean population data, is available in Table S9.

HLA-DRB3*02:02:01G, HLA-DRB4*01:03:01G, and HLA-DRB5*01:02 were the most frequent alleles of *HLA-DRB3*, *HLA-DRB4*, and *HLA-DRB5*, respectively. Additional analyses showed that the previously described linkage between *HLA-DRB3* and *HLA-DR11*, *HLA-DR12*, *HLA-DR13*, *HLA-DR14*, *HLA-DR17*, and *HLA-DR18*; *HLA-DRB4* and *HLA-DR4*, *HLA-DR7*, and *HLA-DR9*; *HLA-DRB5* and *HLA-DR15* and *HLA-DR16*; and none-*HLA3/4/5* and *HLA-DR1*, *HLA-DR8*, and *HLA-DR10* were 100% matched in this study. Detailed information is available in Table S10.

### 3.4. HLA Allele Frequency within Patients with Specific Hematologic Diseases and the General Population

Comparison of HLA frequency between patients with specific hematologic diseases and the general population is shown in Table 5. HLA-A*11:01 and HLA-A*31:01:02:01 were more frequent in AML and BLL patients, respectively, but their frequencies did not differ remarkably from that in the general population (*p* > 0.05). HLA-B*35:01 and HLA-B*40:02:01:01 were two times more frequent in MDS and AA, respectively, than in the normal population (*p* > 0.05). In addition, HLA-C*01:02 and HLA-C*15:02 tended to be more frequent in AML and BLL patients, respectively (*p* > 0.05). Interestingly, patients with MDS were six-times more likely to harbor HLA-DRB1*08:02:01G (*p* < 0.05). HLA-DRB1*14:07:01 was more frequent in AML or MDS patients than in the general Korean population. Similar analyses were also performed based on HLA typing NGS data from another Korean study [6]; however, given the reduced size of the study cohort (*n* = 128), the analysis lacked statistical power and prevented an accurate interpretation of the results.

## 4. Discussion

This study demonstrated a single-center experience on HLA typing using NGS in Korean patients with hematologic disease. Three-field HLA typing with G code was successfully achieved for all loci and known linkage between *HLA-DRB3/4/5* and *HLA-DRB1* was fully matched. In addition, we collected population data on *HLA-DQA1*, *HLA-DPB1*, and *HLA-DPA1* frequency in a Korean population for the first time. Furthermore, we showed that some HLA alleles are increased in various hematologic diseases; in particular, that HLA-DRB1*08:02:01G is significantly increased in MDS patients compared with the general Korean population.

Low-resolution HLA typing continues to be used in the clinic owing to its time-efficient and less expensive features. In contrast, high-resolution HLA typing can overcome the detection limitations of low-resolution HLA typing methods, but it is time-consuming. Nonetheless, innovative strategies using NGS allow a turnaround time for HLA typing of two days [13]. An important advantage of NGS was the introduction of four-field high-resolution HLA typing that can cover the entire targeted genes. In this study, HLA typing data covering 11 loci of HLA genes were reviewed. All data were deemed accurate, and successful HLA typing was achieved until three-field resolution; however, four-field typing showed variable outcomes for the different alleles evaluated, with a relatively low success rate. To date, the reference sequence for introns remains unclear, and the sequence at the end of the untranslated region is also not clear or the depth is low, which can hamper the accuracy of the typing results.

The linkage between *HLA-DRB3/4/5* and *HLA-DRB1* has been widely recognized, and the clinical significance of anti-*HLA-DRB3/4/5* donor-specific antibodies has been suggested [14]. However, no report describing this linkage and the high-resolution frequency of *HLA-DRB3/4/5* in the Korean population was available. Herein, we confirmed that all participants correctly matched their *HLA-DRB1* and *HLA-DRB3/4/5* linkage. Although not absolutely necessary, this result can be useful to predict the outcome of *HLA-DRB3/4/5* and *HLA-DRB1* typing based on each other. The polymorphism and frequency diversity of *HLA-DRB3*, *HLA-DRB4*, and *HLA-DRB5* is presented in Table S10.

In our study, although only patients who met the criteria of HSCT were retrospectively enrolled, *HLA-DPA1*, *HLA-DPB1*, *HLA-DQA1*, *HLA-DQB1*, and *HLA-DRB3/4/5* alleles were shown to be the most prevalent. HLA-DPA1*02:02:02G and HLA-DPA1*01:03:01G showed high frequency (45.25% and 38.24%, respectively). HLA-DRB4*01:03 had the highest frequency in *HLA-DRB3/4/5* 2-field typing (49.20%). This is the first study to explore the typing outcome of *HLA-DPA1*, *HLA-DPB1*, *HLA-DQA1*, and *HLA-DRB3/4/5* using NGS in Koreans. The collected data provide a useful database on the frequency of these genes. Detailed information is available in Tables S6–S10.

HLA typing with NGS is a successful three-field resolution approach, representing a solution to overcome the ambiguity and strength problems with a very low error rate [8,9]. Owing its low error rate, lower costs, and less data ambiguities, NGS approaches have been proposed as an easy and cost-effective method for HLA typing for HSCT [15]. Unlike the SBT or low-resolution typing methods, NGS does not require further analysis to confirm the typed *HLA* alleles. It is worthy of note that HLA typing for HSCT is covered by the National Health Insurance in Korea; thus, HLA typing for five loci (*HLA-A*, *B*, *C*, *DR*, *DQ*) costs approximately $100 via the SBT method and $68 via NGS, which significantly reduces the economic burden to the patient. For the cost-effectiveness of NGS HLA typing, it better to collect several samples and test them at once, so the reporting time is actually delayed by about three to four days compared with that of SBT HLA typing. NGS is not disadvantageous compared with SBT HLA typing concerning long turnaround times because it does not require an additional process to solve ambiguity. In the case of NGS, 50–60 samples can be tested at once in one batch using a Nextseq 550Dx system (Illumina), and it takes about 10 min for each locus in the case of SBT to interpret the results, whereas in the case of NGS, it takes less than 10 min to interpret all 11 loci. Therefore, the NGS method also helps in the efficient use of human resources. Hence, HLA typing using NGS may have enhanced clinical utility; nonetheless, complex and expensive equipment and computer systems are required.

Several studies have suggested an association between HLA and hematologic diseases. For instance, Yoon suggested an association between *HLA-C3* and AML in Koreans [10]; however, the present study showed that HLA-C*03:03 frequencies were higher (but not statistically different) in AML and MDS patients than in the general population. The association between HLA-B*40:02 and AA has been extensively studied in the Japanese population [11,16]. Herein, a two-fold and four-fold increase in AA patients compared with reference data was observed; however, these differences were not statistically significant (*p* = 0.35) due to the small sample size. Moreover, HLA-B*40:02 allele frequency in leukemia patients was lower than in the general population. Notably, HLA-DRB1*14:07:01 was associated with AML/MDS, but this result should be further confirmed in a larger sample size. In particular, HLA-DRB1*14:07 frequency varies in the Korean population between 0.08% and 1.6% [5,6]; hence, further population studies on this allele are needed. Furthermore, HLA-DRB1*08:02:01G was found to be remarkably increased among MDS patients. Importantly, to date, this allele has only been discussed in relation to bucillamine-induced proteinuria in Japanese patients with rheumatoid arthritis [17]; therefore, further studies are warranted to confirm this new association.

There are some limitations to this study. Overall, although we observed possible associations between hematologic malignancies and certain *HLA* alleles, the sample size was small; thus, further studies to confirm the findings reported herein are necessary. Moreover, given the small sample size, some associations may have been missed. Given the retrospective nature of the study, only patients who were scheduled to undergo HSCT were enrolled, and no control-matched population was included. Because HLA typing using NGS started in June 2019 in our hospital, little data was available covering specific diseases at the time of the study. Nonetheless, we are still collecting and reviewing additional data to strengthen our analyses.

## 5. Conclusions

In this study, we described HLA typing results using NGS and investigated the association between *HLA* variability and hematologic malignancies. In summary, we confirmed that NGS makes high-resolution HLA typing possible, which can aid in the selection of suitable donors for HSCT. Furthermore, this study suggests an association between certain *HLA* alleles and hematologic malignancies, requiring further investigation in large cohorts and experiments with certain *HLA* alleles. Lastly, the reported data on NGS-based HLA typing of *HLA-DP*, *HLA-DQ*, *HLA-DRB3*, *HLA-DRB4*, and *HLA-DRB5* provide a foundation to improve the databases on the corresponding locus.

## Figures and Tables

**Table 1:** Demographic features of the patients diagnosed with hematologic diseases.

Parameter.	No. (%)
Sex	
Male	123 (55.7)
Female	98 (44.3)
Age	
0–20	44 (19.9)
21–40	49 (22.2)
41–60	86 (38.9)
≥61	42 (19.0)
Diagnosis	
Acute myeloid leukemia	87 (39.4)
B-cell acute lymphoblastic leukemia	43 (19.5)
Myelodysplastic syndrome	22 (10.0)
Aplastic anemia	14 (6.3)
T-cell lymphoma	13 (5.9)
Chronic myeloid leukemia	7 (3.2)
Other hematologic disease	35 (15.8)

**Table 2:** Success rate (%) of high-resolution HLA typing.

Resolution	*A*	*B*	*C*	*DRB1*	*DRB3/4/5*	*DQA1*	*DQB1*	*DPA1*	*DPB1*
2-field	100.00	100.00	100.00	100.00	100.00	96.83	100.00	100.00	99.10
3-field	100.00	100.00	100.00	100.00	100.00	96.83	100.00	100.00	99.10
4-field	99.10	88.24	73.53	64.93	54.89	92.31	22.62	16.29	7.92

**Table 3:** Summary of *HLA-A*, *HLA-B*, and *HLA-C* typing (2-field) results.

*HLA-A*	This Study	Ref. Data		*HLA-B*	This Study	Ref. Data		*HLA-C*	This Study	Ref. Data	
	Total	Severance Database	Choe et al. [6]		Total	Severance Database	Choe et al. [6]		Total	Severance Database	Choe et al. [6]
	(*n* = 221)	(*n* = 729)	(*n* = 128)		(*n* = 221)	(*n* = 747)	(*n* = 128)		(*n* = 221)	(*n* = 712)	(*n* = 128)
01:01	1.13	1.58	2.3	07:02	2.94	3.01	2.7	01:02	19.91	16.29	19.9
02:01	14.94	15.91	14.5	07:05	0.68	0.40	0.4	01:03	0.45	0.56	0.4
02:03	0.45	0.34	0.8	08:01		0.54	0.8	01:09		0.07	
02:05		0.07		13:01	2.04	2.28	4.3	01:135		0.07	
02:06	10.41	10.08	9.8	13:02	2.27	2.48	2.7	02:02	0.68	0.91	1.2
02:07	2.26	3.91	3.5	14:01	1.36	0.80	0.8	03:02	5.43	6.81	6.7
02:10	0.23	0.07	0.4	14:02		0.07		03:03	11.76	10.81	9.4
02:41	0.23			15:01	10.41	10.17	10.2	03:04	8.14	9.41	11.3
02:53			0.4	15:02	1.13	0.60		03:158		0.07	
03:01	2.71	2.81	1.2	15:07	0.68	1.00	0.8	03:43		0.07	
03:02		0.27		15:08		0.07		04:01	6.11	7.37	9.0
11:01	13.80	10.08	13.3	15:11	0.23	1.87	0.4	04:82	0.68		
11:02	0.45	0.34	1.6	15:18	1.81	1.00	0.4	05:01	2.04	1.54	1.2
11:19		0.07		15:25		0.07		06:02	4.08	4.49	3.1
11:20		0.07	0.4	15:27	0.68	0.40		07:02	7.91	8.57	8.2
24:02	21.95	20.51	19.9	15:38		0.27	0.8	07:04	1.58	1.05	0.4
24:08		0.07	0.4	18:01	0.23	0.13		07:06/07:01		3.02	
24:10		0.14		18:02		0.13		07:06	4.75		3.1
24:20	0.23	0.21	0.4	27:04	0.23	0.07	0.4	08:01/08:22	6.12	7.09	5.5
26:01	3.85	4.39	3.5	27:05	3.39	2.61	4.3	08:02	1.36	0.91	
26:02	0.68	1.99	3.5	27:20	0.23			08:03	0.45	0.98	0.8
26:03	1.81	0.96	0.4	35:01	6.57	4.75	7.4	08:22	0.45		0.8
26:10			0.4	35:03	0.45	0.40		12:02	2.49	2.53	3.5
29:01	0.68	0.48	0.8	37:01	1.36	1.41	0.4	12:03	0.68	0.49	0.4
29:02		0.21		38:01		0.27		14:02	8.14	7.23	7.8
30:01	2.26	2.61	2.0	38:02	0.68	1.14	0.8	14:03	3.39	6.53	5.5
30:04	1.58	1.03	0.8	39:01	0.68	1.07	1.2	15:02	2.71	2.25	0.4
31:01	7.01	4.66	4.3	39:04	0.68			15:04		0.14	
31:11		0.07		40:01	4.97	3.68	3.1	15:05	0.68	0.42	0.4
32:01	0.23	0.62		40:02	2.94	5.09	2.3	16:01		0.14	
33:01		0.07		40:03	0.23	0.20	0.8	16:02		0.07	
33:03	12.90	15.91	15.6	40:06	3.40	3.82	2.7	17:01		0.07	
33:25	0.23			41:01		0.07					
68:01		0.27		44:02	2.04	1.61	1.2				
68:02		0.21		44:03	8.14	9.57	8.6				
				46:01	5.21	4.69	5.5				
				47:01	0.23	0.13					
				48:01	2.49	3.55	3.9				
				48:03		0.07					
				48:47	0.23						
				50:01		0.27					
				51:01	10.86	9.77	9				
				51:02	0.68	0.33	0.4				
				52:01	2.27	2.48	3.1				
				54:01	5.66	5.22	7				
				55:01	0.23	0.07	0.4				
				55:02	2.27	1.54	3.2				
				55:04		0.07					
				55:07		0.07					
				56:01	0.45	0.33	0.4				
				57:01	0.23	0.40	0.4				
				58:01	5.43	6.63	5.5				
				59:01	2.26	2.14	2				
				67:01	1.13	1.20	2.4				

**Table 4:** Summary of *HLA-DRB1* and *HLA-DQB1* typing (2-field) results.

*HLA-DRB1*	This Study	Ref. Data		*HLA-DQB1*	This Study	Ref. Data
	Total	Severance Database	Choe et al. [6]		Total	In et al. [5]
	(*n* = 221)	(*n* = 761)	(*n* = 128)		(*n* = 221)	(*n* = 613)
01:01	6.33	5.78	5.9	02:01	1.81	2.12
01:02		0.07		02:02	6.79	6.69
03:01	1.81	2.04	2.7	03:01	14.25	14.03
04:01	1.13	0.85		03:02	9.96	9.62
04:03	2.94	4.07	1.6	03:03	11.76	11.17
04:04	0.90	1.18	2.3	04:01	10.64	8.81
04:05	11.08	9.26	8.2	04:02	3.85	3.92
04:06	6.33	5.19	6.3	05:01	8.37	8.97
04:07		0.26	0.4	05:02	2.71	2.12
04:10	1.13	0.33	1.6	05:03	3.85	4.49
07:01	7.47	6.31	5.9	05:10	0.23	
08:01		0.20		06:01	9.28	9.38
08:02	1.36	1.12	2.0	06:02	8.14	7.75
08:03	7.47	5.98	9.4	06:03	1.36	1.63
09:01	10.18	11.89	10.2	06:04	3.39	5.06
10:01	1.58	1.25		06:09	3.62	4.24
11:01	4.98	4.53	5.5			
11:04		0.07				
12:01	4.75	4.34	4.7			
12:02	2.94	4.20	5.1			
12:05		0.07				
13:01	1.36	2.10	2.7			
13:02	7.00	9.33	7.4			
14:02	0.23	0.07				
14:03	1.36	0.85	0.8			
14:05	1.36	2.56	3.1			
14:06		0.66				
14:07	1.13	0.33	1.6			
14:10	0.23	0.07				
14:12		0.13	0.4			
14:54	2.26	2.83	1.6			
15:01	8.83	8.21	7.0			
15:02	2.71	2.76	2.7			
16:01		0.07				
16:02	1.13	1.05	1.2			

**Table 5:** Comparison of specific allele frequencies between patients with hematologic diseases and the general Korean population.

HLA Typing	Diagnosis	Population (%)	Severance (%)	*p*-Value
HLA-A*11:01	AML	15.52	10.08	0.12
HLA-A*31:01:02:01	BLL	9.30	4.66	0.17
HLA-B*35:01	MDS	11.36	4.75	0.16
HLA-B*40:02:01:01	AA	10.71	5.09	0.35
HLA-C*01:02	AML	22.41	16.29	0.15
HLA-C*15:02	BLL	5.81	2.25	0.14
HLA-DRB1*08:02:01G	MDS	6.82	1.12	0.02
HLA-DRB1*14:07:01	AML	1.72	0.33	0.07
HLA-DRB1*14:07:01	MDS	2.27	0.33	0.15

Abbreviations: AA, aplastic anemia; AML, acute myeloid leukemia; BLL, B lymphoblastic leukemia; MDS, myelodysplastic syndrome.

## Data Availability

The data presented in this study are available on request from the corresponding author. The data are not publicly available due to patients’ privacy.

## Supplementary Materials

The following supporting information can be downloaded at: https://www.mdpi.com/article/10.3390/diagnostics12051074/s1, Table S1: TypeStream Visual analysis parameter configuration; Table S2: Detailed *HLA-A* typing results (shown as percentage [%]); Table S3: Detailed *HLA-B* typing results (shown as percentage [%]); Table S4: Detailed *HLA-C* typing results (shown as percentage [%]); Table S5: Detailed *HLA-DRB1* typing results (shown as percentage [%]); Table S6: Detailed *HLA-DPA1* typing results (shown as percentage [%]); Table S7: Detailed *HLA-DPB1* typing results (shown as percentage [%]); Table S8: Detailed *HLA-DQA1* typing results (shown as percentage [%]); Table S9: Detailed *HLA-DQB* typing results (shown as percentage [%]); Table S10: Detailed *HLA-DRB345* typing results (shown as percentage [%]).
