# Supplementary material for: A Single-Center Experience on HLA Typing with 11 Loci Next Generation Sequencing in Korean Patients with Hematologic Disease
Source: Diagnostics (Basel). 2022 Apr 25;12(5):1074. doi: 10.3390/diagnostics12051074 (PMC9139519; doi:10.3390/diagnostics12051074)
Supplement: Supplementary file 1 [file diagnostics-12-01074-s001.zip › diagnostics-1661594-supplementary.pdf]

Supplementary Table S1. TypeStream Visual analysis parameter configuration

Min read length	100
Max insertion	3
Max deletion	3
Max mismatch bases	5
Min base read depth	20
Min valid read	500
High background cutoff (%)	20
Min hetero allele balance	10
Max read for typing	300000

Supplementary Table S2. Detailed HLA-A typing results (shown as percentage [%])

HLA-A*	This study						Ref. data	
	AML	BLL	MDS	TCL	AA	Total	Severance	Choe et al.
	(n = 87)	(n = 43)	(n = 22)	(n = 13)	(n = 14)	(n = 221)	(n = 729)	(n = 128)
**01:01**							1.58	
**01:01:01:01**	1.72			3.85		1.13		2.3
**02:01**							15.91	
**02:01:01:01**	14.94	11.63	18.18	15.38	14.29	14.71		14.5
**02:01:01:97**	0.57					0.23		
**02:03**							0.34	
**02:03:01**	0.57			3.85		0.45		0.8
**02:05**							0.07	
**02:06**							10.08	
**02:06:01:01**	9.2	8.14	9.09	11.54	7.14	9.73		9.8
**02:06:01:04**		1.16	2.27			0.68		
**02:07**							3.91	3.5
**02:07:01:01**	1.72	1.16	4.55		3.57	2.26		
**02:10**	0.57					0.23	0.07	0.4
**02:41**		1.16				0.23		
**02:53**								0.4
**03:01**							2.81	
**03:01:01:01**	1.72	2.33		7.69		2.26		1.2
**03:01:01G**	1.15					0.45		
**03:02**							0.27	
**11:01**							10.08	
**11:01:01:01**	14.37	12.79	6.82	7.69	17.86	13.35		13.3
**11:01:01G**	1.15					0.45		
**11:02**							0.34	1.6
**11:02:01:01**		1.16	2.27			0.45		
**11:19**							0.07	
**11:20**							0.07	0.4
**24:02**							20.51	
**24:02:01:01**	22.99	25.58	18.18	19.23	14.29	21.95		19.5
**24:02:01:02L**								0.4
**24:08**							0.07	0.4
**24:10**							0.14	
**24:20**							0.21	0.4
**24:20:01:02**						0.23		
**26:01**							4.39	
**26:01:01:01**	1.72	2.33	2.27	7.69	7.14	3.62		3.5
**26:01:01:12**	0.57					0.23		

**26:02**							1.99	
**26:02:01**	0.57	1.16			3.57	0.68		3.5
**26:03**							0.96	0.4
**26:03:01:01**	1.72	3.49			3.57	1.58		
**26:03:01:02**	0.57					0.23		
**26:10**								0.4
**29:01**							0.48	
**29:01:01:01**	0.57	1.16		3.85		0.68		0.8
**29:02**							0.21	
**30:01**							2.61	2
**30:01:01:01**	1.72	2.33	9.09		3.57	2.26		
**30:04**							1.03	0.8
**30:04:01:01**	1.15	2.33		3.85		1.58		
**31:01**							4.66	
**31:01:02:01**	6.9	9.3	11.36	3.85	7.14	7.01		4.3
**31:11**							0.07	
**32:01**							0.62	
**32:01:01:01**	0.57					0.23		
**33:01**							0.07	
**33:03**							15.91	15.6
**33:03:01:01**	13.22	11.63	15.91	11.54	17.86	12.9		
**33:25**		1.16				0.23		
**68:01**							0.27	
**68:02**							0.21	

Supplementary Table S3. Detailed HLA-B typing results (shown as percentage [%])

**HLA-B***	This study						Ref. data	
	AML (n = 87)	BLL (n = 43)	MDS (n = 22)	TCL (n = 13)	AA (n = 14)	Total (n = 221)	Severance (n = 747)	Choe et al. (n = 128)
**07:02**							3.01	
**07:02:01:01**	2.3	3.49				2.94		2.7
**07:05**							0.4	
**07:05:01G**	0.57	1.16		3.85		0.68		0.4
**08:01**							0.54	0.8
**13:01**							2.28	4.3
**13:01:01:01**	1.72	1.16			3.57	2.04		
**13:02**							2.48	2.7
**13:02:01:01**	1.72	2.33	9.09			2.04		
**13:02:01G**	0.57					0.23		
**14:01**							0.8	0.8
**14:01:01:01**	1.15	3.49		3.85		1.36		
**14:02**							0.07	
**15:01**							10.17	
**15:01:01:01**	12.64	6.98	11.36		14.29	10.41		10.2
**15:02**							0.6	
**15:02:01:01**	1.72	1.16				1.13		
**15:07**							1	
**15:07:01:01**	1.15					0.68		0.8
**15:08**							0.07	
**15:11**							1.87	0.4
**15:11:01**		1.16				0.23		
**15:18**							1	
**15:18:01:02**	1.15	3.49		7.69		1.81		0.4
**15:25**							0.07	
**15:27**							0.4	
**15:27:01**		2.33				0.68		
**15:38**							0.27	0.8
**18:01**							0.13	
**18:01:01G**						0.23		

**18:02**							0.13	
**27:04**							0.07	
**27:04:01**		1.16				0.23		0.4
**27:05**							2.61	4.3
**27:05:02:05**	4.02	1.16	2.27	3.85	3.57	3.39		
**27:20**			2.27			0.23		
**35:01**							4.75	
**35:01:01:02**	5.75	2.33	9.09	3.85	3.57	4.98		6.6
**35:01:01:05**						0.23		
**35:01:01:06**								0.8
**35:01:01:19**						0.23		
**35:01:01G**	1.15	1.16	2.27			1.13		
**35:03**							0.4	
**35:03:01:01**	1.15					0.45		
**37:01**							1.41	0.4
**37:01:01:01**	1.72			3.85		1.13		
**37:01:01G**						0.23		
**38:01**							0.27	
**38:02**							1.14	0.8
**38:02:01:01**	0.57	1.16		3.85		0.68		
**39:01**							1.07	1.2
**39:01:01:03**		1.16				0.45		
**39:01:01G**					3.57	0.23		
**39:04**	0.57					0.68		
**40:01**							3.68	
**40:01:02:11**	0.57					0.45		
**40:01:02G**	5.17	5.81	2.27	3.85	7.14	4.52		3.1
**40:02**							5.09	
**40:02:01:01**	1.15	1.16	2.27	3.85	10.71	2.71		2.3
**40:02:01G**						0.23		
**40:03**							0.2	0.8
**40:03:01:02**	0.57					0.23		
**40:06**							3.82	2.7
**40:06:01:12**	2.87	2.33	6.82	3.85	3.57	3.17		
**40:06:01G**				3.85		0.23		
**41:01**							0.07	
**44:02**							1.61	
**44:02:01:01**	2.3	2.33		7.69		2.04		1.2
**44:03**							9.57	
**44:03:01:10**	4.02		6.82	3.85	3.57	3.39		
**44:03:01G**								5.5
**44:03:02:01**	4.6	6.98	2.27	3.85		4.52		
**44:03:02G**						0.23		3.1
**46:01**							4.69	5.5
**46:01:01:01**	4.02	6.98	9.09		7.14	4.98		
**46:01:01G**	0.57					0.23		
**47:01**							0.13	
**47:01:01:03**					3.57	0.23		
**48:01**							3.55	3.9
**48:01:01:01**	2.87	1.16		3.85	7.14	2.26		
**48:01:01:02**						0.23		
**48:03**							0.07	
**48:47**		1.16				0.23		
**50:01**							0.27	
**51:01**							9.77	
**51:01:01:01**	8.05	6.98	11.36	11.54	7.14	7.92		6.6
**51:01:01:36**		2.33		3.85		0.68		
**51:01:01G**	1.72	3.49	2.27	3.85		2.26		2.4
**51:02**							0.33	
**51:02:01G**		2.33	2.27			0.68		0.4
**52:01**							2.48	
**52:01:01:02**	2.3	2.33	4.55		3.57	2.04		3.1
**52:01:01G**	0.57					0.23		
**54:01**							5.22	7
**54:01:01:01**	5.17	4.65	6.82	7.69	3.57	5.43		
**54:01:01G**	0.57					0.23		
**55:01**							0.07	0.4

**55:01:01:01**		1.16				0.23		
**55:02**							1.54	
**55:02:01:01**								0.8
**55:02:01:02**		1.16				0.68		2
**55:02:01:03**	1.72				3.57	1.36		
**55:02:01:04**	0.57					0.23		0.4
**55:04**							0.07	
**55:07**							0.07	
**56:01**							0.33	
**56:01:01:04**	0.57	1.16				0.45		0.4
**57:01**							0.4	0.4
**57:01:01:01**	0.57					0.23		
**58:01**							6.63	
**58:01:01:01**								0.4
**58:01:01:03**	5.17	6.98	6.82	3.85	7.14	5.2		5.1
**58:01:01G**						0.23		
**59:01**							2.14	
**59:01:01:01**	3.45	1.16		3.85	3.57	2.26		2
**67:01**							1.2	
**67:01:01**	1.15	2.33				0.9		1.6
**67:01:02**		1.16				0.23		0.8

Supplementary Table S4. Detailed HLA-C typing results (shown as percentage [%])

HLA-C*	This study						Ref. data	
	AML	BLL	MDS	TCL	AA	Total	Severance	Choe et al.
	(n = 87)	(n = 43)	(n = 22)	(n = 13)	(n = 14)	(n = 221)	(n = 712)	(n = 128)
**01:02**							16.29	19.9
**01:02:01:01**	15.52	11.63	13.64	7.69	17.86	13.35		
**01:02:01:05**	6.32	4.65	6.82	7.69	3.57	5.66		
**01:02:01:08**	0.57					0.45		
**01:02:01G**				3.85		0.45		
**01:03**	0.57					0.45	0.56	0.4
**01:09**							0.07	
**01:135**							0.07	
**02:02**							0.91	
**02:02:02G**	1.15					0.68		1.2
**03:02**							6.81	
**03:02:02:01**								6.3
**03:02:02:03**	0.57	1.16				0.45		0.4
**03:02:02:05**	4.6	5.81	6.82	3.85	3.57	4.75		
**03:02:02G**					3.57	0.23		
**03:03**							10.81	
**03:03:01:01**	13.22	9.3	15.91	7.69	14.29	11.76		9.4
**03:04**							9.41	
**03:04:01G**	6.9	4.65	2.27	7.69	14.29	8.14		11.3
**03:158**							0.07	
**03:43**							0.07	
**04:01**							7.37	
**04:01:01G**	5.17	5.81	2.27		10.71	6.11		9
**04:82**	1.15				3.57	0.68		
**05:01**							1.54	
**05:01:01:02**	2.3	2.33		7.69		2.04		1.2
**06:02**							4.49	
**06:02:01:01**	4.02	2.33	9.09	3.85	3.57	3.85		3.1
**06:02:01G**	0.57					0.23		
**07:02**							8.57	
**07:02:01:01**	3.45	4.65		3.85		2.94		5.5
**07:02:01:03**	2.3	3.49				2.71		2.7
**07:02:01:15**	0.57	3.49			3.57	1.58		
**07:02:01:52**	0.57					0.23		
**07:02:01G**		1.16	2.27			0.45		
**07:04**							1.05	
**07:04:01G**	1.15	4.65		3.85		1.58		0.4
**07:06/07:01**							3.02	
**07:06**								3.1
**07:06:01:01**	4.6	6.98	2.27	3.85		4.75		

**08:01/08:22**							7.09	5.5
**08:01:01:01**	5.75	4.65	6.82	7.69	7.14	5.66		
**08:01:01:08**			2.27			0.23		
**08:01:01G**						0.23		
**08:02**							0.91	
**08:02:01:02**	1.15	3.49		3.85		1.36		
**08:03**							0.98	
**8:03:01**	0.57	1.16				0.45		0.8
**08:22**								0.8
**08:22:01:01**	0.57					0.45		
**12:02**							2.53	
**12:02:02:01**	2.3	3.49	6.82		3.57	2.49		3.5
**12:03**							0.49	
**12:03:01:01**	0.57					0.45		0.4
**12:03:01G**	0.57					0.23		
**14:02**							7.23	
**14:02:01G**	6.9	8.14	9.09	15.38	7.14	8.14		7.8
**14:03**							6.53	5.5
**14:03:01:01**	4.02		6.82	3.85	3.57	3.39		
**15:02**							2.25	
**15:02:01:01**	1.15	4.65	6.82	3.85		2.26		
**15:02:01:03**								0.4
**15:02:01:06**	0.57	1.16				0.45		
**15:04**							0.14	
**15:05**							0.42	0.4
**15:05:02:01**	0.57	1.16		3.85		0.68		
**16:01**							0.14	
**16:02**							0.07	
**17:01**							0.07	

Supplementary Table S5. Detailed HLA-DRB1 typing results (shown as percentage [%])

HLA-DRB1*	This study						Ref. data	
	AML	BLL	MDS	TCL	AA	Total	Severance	Choe et al.
	(n = 87)	(n = 43)	(n = 22)	(n = 13)	(n = 14)	(n = 221)	(n = 761)	(n = 128)
**01:01**							5.78	5.9
**01:01:01G**	5.17	4.65	2.27	3.85	3.57	6.33		
**01:02**							0.07	
**03:01**							2.04	
**03:01:01:01**								2.7
**03:01:01:03**	2.3	1.16	2.27			1.58		
**03:01:01G**		1.16				0.23		
**04:01**							0.85	
**04:01:01:01**	0.57	1.16		7.69		0.9		
**04:01:01:03**						0.23		
**04:03**							4.07	1.6
**04:03:01:01**	2.3	4.65	2.27	7.69		2.94		
**04:04**							1.18	
**04:04:01**		3.49		3.85		0.9		2.3
**04:05**							9.26	8.2
**04:05:01:01**	2.87	1.16	2.27		3.57	2.26		
**04:05:01:04**	10.34	9.3	6.82	11.54	10.71	8.82		
**04:06**							5.19	
**04:06:01**	5.75	4.65	4.55	3.85	10.71	6.33		6.3
**04:07**							0.26	0.4
**04:10**							0.33	
**04:10:01**		1.16	2.27		7.14	1.13		0.8
**04:10:03**								0.8
**07:01**							6.31	
**07:01:01:01**	7.47	8.14	11.36	7.69		7.47		5.1
**07:01:01:02**								0.8
**08:01**							0.2	
**08:02**							1.12	
**08:02:01G**	0.57	2.33	6.82			1.36		2
**08:03**							5.98	9.4
**08:03:02:02**	6.32	9.3	6.82	3.85	3.57	6.79		
**08:03:02:03**	0.57	1.16				0.45		
**08:03:02G**				3.85		0.23		

**09:01**							11.89	
**09:01:02G**	8.05	8.14	11.36	7.69	21.43	10.18		10.2
**10:01**							1.25	
**10:01:01:03**	2.3			3.85		1.58		
**11:01**							4.53	
**11:01:01:01**	0.57					0.45		5.5
**11:01:01:03**	2.87				3.57	1.36		
**11:01:01:04**	2.3		2.27		3.57	1.36		
**11:01:01G**	1.72	2.33		7.69		1.81		
**11:04**							0.07	
**12:01**							4.34	
**12:01:01G**	5.17	5.81	4.55		3.57	4.75		4.7
**12:02**							4.2	5.1
**12:02:01:04**	0.57					0.23		
**12:02:01:06**	1.72	1.16	2.27	3.85		1.81		
**12:02:01G**	0.57	2.33			3.57	0.9		
**12:05**							0.07	
**13:01**							2.1	
**13:01:01:01**		1.16			3.57	0.68		2.7
**13:01:01G**	1.15	1.16				0.68		
**13:02**							9.33	7.4
**13:02:01:01**	1.15	2.33			10.71	1.81		
**13:02:01:02**	0.57		4.55	3.85		1.58		
**13:02:01:03**	0.57	2.33	2.27			0.9		
**13:02:01G**	4.02		4.55	3.85	3.57	2.71		
**14:02**							0.07	
**14:02:01:01**	0.57					0.23		
**14:03**							0.85	0.8
**14:03:01**	0.57	3.49				1.36		
**14:05**							2.56	3.1
**14:05:01:01**		1.16				0.23		
**14:05:01:02**	1.15					0.45		
**14:05:01:03**	0.57					0.23		
**14:05:01G**		1.16	2.27			0.45		
**14:06**							0.66	
**14:07**							0.33	
**14:07:01**	1.72	1.16	2.27			1.13		1.6
**14:10**	0.57					0.23	0.07	
**14:12**							0.13	0.4
**14:54**							2.83	1.6
**14:54:01:03**	2.87					1.58		
**14:54:01:08**	0.57					0.23		
**14:54:01G**	0.57	1.16				0.45		
**15:01**							8.21	
**15:01:01:01**	2.87		2.27	7.69	3.57	2.94		
**15:01:01:02**		1.16				0.23		
**15:01:01:03**	3.45	5.81	9.09			4.07		7
**15:01:01:04**						0.23		
**15:01:01:05**	0.57					0.23		
**15:01:01G**	2.3					1.13		
**15:02**							2.76	2.7
**15:02:01G**	2.87	2.33	4.55	3.85	3.57	2.71		
**16:01**							0.07	
**16:02**							1.05	1.2
**16:02:01G**	1.15	2.33		3.85		1.13		

Supplementary Table S6. Detailed HLA-DPA1 typing results (shown as percentage [%])

**HLA-DPA1***	AML (n=87)	BLL (n=43)	MDS (n=22)	TCL (n=13)	AA (n=14)	Total (n=221)
**01:03:01:01**			2.27			0.23
**01:03:01:04**	3.45	1.16	2.27			2.26
**01:03:01:05**	0.57			3.85		0.45
**01:03:01:07**			2.27			0.23
**01:03:01:16**		1.16		3.85		0.45
**01:03:01:20**	1.72	3.49	2.27			2.49
**01:03:01G**	37.36	43.02	40.91	30.77	46.43	38.24

**01:30**	0.57					0.45
**02:01:01:01**	3.45					1.36
**02:01:01:02**	2.30	3.49	2.27	3.85	7.14	2.71
**02:01:01:03**	1.15		4.55	3.85		1.13
**02:01:01:06**	1.72	2.33	2.27		3.57	2.26
**02:01:01:13**	0.57	1.16	2.27		7.14	1.36
**02:01:01:14**		1.16				0.23
**02:01:01:17**			2.27			0.23
**02:02:02G**	46.55	43.02	36.36	53.85	35.71	45.25
**02:07:01:01**						0.23
**02:07:01G**						0.23
**04:01:01:02**	0.57					0.23

Supplementary Table S7. Detailed HLA-DPB1 typing results (shown as percentage [%])

**HLA-DPB1***	AML (n=87)	BLL (n=43)	MDS (n=22)	TCL (n=13)	AA (n=14)	Total (n=221)
**02:01:02:01**		1.16				0.23
**02:01:02:11**	0.59					0.23
**02:01:02:26**						0.23
**02:01:02:36**	1.76		2.27			0.91
**02:01:02:45**	0.59					0.23
**02:01:02G**	23.53	27.91	38.64	23.08	21.43	26.26
**02:02:01:01**	4.71	1.16	2.27			2.74
**02:02:01G**	0.59	4.65	4.55	7.69	3.57	2.97
**03:01:01G**	3.53	3.49		3.85	10.71	3.20
**04:01:01:02**		1.16	2.27			0.46
**04:01:01G**	5.29	2.33	4.55	11.54	3.57	5.25
**04:02:01G**	8.82	13.95	4.55	3.85	7.14	9.59
**05:01:01:01**	0.59					0.23
**05:01:01G**	36.47	36.05	31.82	26.92	32.14	36.07
**09:01:01**	1.76	1.16				0.91
**09:01:01G**	0.59		2.27		3.57	0.68
**10:01:01G**						0.23
**100:01**	0.59					0.23
**13:01:01G**	8.24	5.81	2.27	11.54	7.14	6.39
**14:01:01G**		1.16		3.85	3.57	0.68
**17:01:01:01**	1.18		4.55	3.85		1.14
**21:01**	0.59					0.23
**29:01:01G**	0.59			3.85	3.57	0.68
**45:01**					3.57	0.23

Supplementary Table S8. Detailed HLA-DQA1 typing results (shown as percentage [%])

**HLA-DQA1***	AML (n=87)	BLL (n=43)	MDS (n=22)	TCL (n=13)	AA (n=14)	Total (n=221)
**01:01:01:01**	4.27	4.65	2.38	3.85	3.57	6.07
**01:01:01:03**	0.61					0.23
**01:01:01:07**	0.61			3.85		0.47
**01:02:01:01**	8.54	5.81	7.14	3.85	3.57	7.24
**01:02:01:03**		1.16	4.76			0.93
**01:02:01:04**	1.83	4.65	2.38	3.85	7.14	2.57
**01:02:01:08**	3.66		7.14	3.85	3.57	3.50
**01:02:01G**	1.83		2.38	3.85	3.57	1.64
**01:02:02:01**	1.22	2.33		3.85		1.17
**01:03:01:01**	2.44	2.33	4.76		3.57	2.34
**01:03:01:02**	1.22	1.16			3.57	1.17
**01:03:01:04**	3.05	3.49		3.85	3.57	3.50
**01:03:01:07**	3.05	4.65	4.76			2.57
**01:03:01:08**		2.33				0.47
**01:04:01:01**	8.54	2.33	2.38			4.21
**01:04:01:02**		1.16				0.23
**01:04:01:04**		1.16				0.47
**01:05:01:01**	2.44			3.85		1.64
**02:01:01:01**	7.32	8.14	11.90	7.69		7.48

**03:01:01:01**	7.93	9.30	7.14	11.54	14.29	9.81
**03:02:01:01**	4.27	8.14	11.90	3.85	25.00	8.41
**03:02:01:02**	3.66	1.16	2.38	3.85		2.57
**03:03:01:02**		1.16	2.38			0.70
**03:03:01:03**	14.02	10.47	9.52	11.54	10.71	11.21
**03:03:01:06**					7.14	0.47
**03:03:01G**	0.61	4.65		11.54		2.10
**04:01:01:02**		2.33	4.76			0.93
**04:01:01:03**	0.61		2.38			0.47
**05:01:01:02**						0.23
**05:01:01:03**	1.83	2.33	2.38			1.40
**05:03:01:02**	0.61	2.33				0.93
**05:05:01:01**		1.16				0.23
**05:05:01:02**	6.10	1.16	2.38	7.69	7.14	4.21
**05:05:01:03**	0.61					0.23
**05:05:01:18**		1.16				0.70
**05:05:01:20**	3.05					1.17
**05:06:01G**	1.83	1.16				0.93
**05:07**	0.61	1.16				0.70
**05:08**		2.33	2.38			0.93
**05:09**	0.61					0.23
**06:01:01:01**	1.83	2.33	2.38	3.85	3.57	1.87
**06:01:01:02**	0.61	1.16				0.93
**06:01:01:03**	0.61	1.16		3.85		0.70

Supplementary Table S9. Detailed HLA-DQB typing results (shown as percentage [%])

HLA-DQB1*	AML (n=87)	BLL (n=43)	MDS (n=22)	TCL (n=13)	AA (n=14)	Total (n=221)	Roh et al. (n=613)
**02:01**							2.12
02:01:01G	2.30	2.33	2.27			1.81	
**02:02**							6.69
02:02:01G	5.75	8.14	11.36	7.69		6.79	
**03:01**							14.03
03:01:01G	16.09	15.12	6.82	23.08	10.71	14.25	
**03:02**							9.62
03:02:01:02	0.57					0.23	
03:02:01G	7.47	10.47	6.82	11.54	14.29	9.73	
**03:03**							11.17
03:03:02:01	1.72					0.68	
03:03:02G	7.47	9.30	13.64	7.69	25.00	10.63	
03:05:01	0.57					0.23	
03:14:01	0.57					0.23	
**04:01**							8.81
04:01:01:01	12.07	10.47	9.09	11.54	10.71	10.41	
04:01:01:03	0.57					0.23	
**04:02**							3.92
04:02:01:04		1.16				0.23	
04:02:01:06		4.65				1.13	
04:02:01:07	0.57	1.16	6.82			1.13	
04:02:01G	1.15		2.27	3.85	7.14	1.36	
**05:01**							8.97
05:01:01G	7.47	4.65	2.27	7.69	3.57	7.92	
05:01:24G	0.57			3.85		0.45	
05:02							2.12
05:02:01G	3.45	3.49		3.85		2.71	
**05:03**							4.49
05:03:01:04	2.30					0.90	
05:03:01G	4.60	3.49	4.55			2.94	
**05:10**	0.57					0.23	
**06:01**							9.38
06:01:01G	8.62	11.63	11.36	3.85	7.14	9.28	
**06:02**							7.75
06:02:01G	8.05	6.98	11.36	7.69	3.57	8.14	
**06:03**							1.63
06:03:01G	1.15	2.33			3.57	1.36	

**06:04**							5.06
06:04:01:01	3.45		6.82	3.85	3.57	3.39	
**06:09**							4.24
06:09:01:01	2.87	4.65	4.55	3.85	10.71	3.62	

Supplementary Table S10. Detailed HLA-DRB345 typing results (shown as percentage [%])

HLA	AML (n=87)	BLL (n=43)	MDS (n=22)	TCL (n=13)	AA (n=14)	Total (n=221)
**DRB3***						
01:01:02:04	0.68	2.82				0.82
01:01:02:05	4.73	7.04	5.41		3.85	4.89
01:01:02G	2.03	2.82			3.85	2.45
01:01:05		1.41				0.54
02:02:01:02	1.35	1.41				0.82
02:02:01:04	1.35	1.41				1.09
02:02:01:06	1.35	1.41			3.85	1.09
02:02:01:09	1.35	2.82			3.85	1.36
02:02:01:10	6.08	1.41				2.99
02:02:01:12						0.27
02:02:01:13	1.35	1.41	2.70			1.09
02:02:01:15	0.68					0.27
02:02:01G	8.78	1.41	10.81	9.09	3.85	6.25
03:01:01:03	3.38	5.63	5.41	4.55	7.69	4.08
03:01:01G	4.05		8.11	4.55	3.85	4.08
03:01:03:01	1.35	1.41				0.82
03:01:03:02	2.03	2.82		4.55	3.85	2.45
**DRB4***						
01:02	0.68	1.41		4.55		1.09
01:03:01:01						0.27
01:03:01:02N	2.03					0.82
01:03:01:04	2.70	2.82		4.55		2.99
01:03:01G	30.41	35.21	35.14	31.82	34.62	32.07
01:03:02	7.43	11.27	13.51	18.18	23.08	11.96
01:04	2.70	2.82		4.55		2.99
01:01:01:02	9.46	8.45	13.51	9.09	3.85	9.24
01:01:01:03						0.27
01:01:01:04	0.68					0.82
01:01:01G	0.68					0.27
**DRB5***						
01:02	9.46	8.45	13.51	9.09	3.85	9.24
02:02:01	1.35	2.82		4.55		1.36
